# Continuing Professional Development ‐ Medical Imaging

**DOI:** 10.1002/jmrs.795

**Published:** 2024-05-12

Maximise your CPD by reading the following selected article and answer the five questions. Please remember to self‐claim your CPD and retain your supporting evidence. Answers will be available via the QR code and published in JMRS – Volume 71, Issue 4 December 2024.

## Medical Imaging – Original Article

### A wellbeing podcast for diagnostic radiography students

Girard E, Punch A, Jimenez Y. (2024) *J Med Radiat Sci*. https://doi.org/10.1002/jmrs.785

1. Which of the following statements best describes a podcast?
  - Research articles available as audio resources.
  - Video content accessible on smartphones or computers.
  - Episodes of audio content accessible on streaming applications.
  - Live radio broadcasts on smartphones or computers.
2. According to this article, which of the following may be a benefit of a podcast series focused on wellbeing topics for radiography students during clinical placements?
  - Provides theoretical knowledge from radiography experts.
  - Offers practical training exercises for radiography techniques.
  - Facilitates networking opportunities with other radiography students.
  - Facilitates sharing of insights from various experiences, including those of students and qualified staff.
3. According to the study findings, which of the following is a limitation of surveys as a data collection tool for the evaluation of podcasts?
  - Lack of interest in the podcast content.
  - Technical difficulties with accessing the survey.
  - Simultaneous engagement in other activities while listening to the podcast.
  - Insufficient promotion of the survey to radiography students.
4. According to the study findings and other literature, how do students perceive the impact of the podcast series on their relationships with peers? Students:
  - Believe the podcast series encourages healthy competition among peers.
  - Feel the podcast series fosters relatability and connectedness among peers.
  - Find the podcast series isolates them from their peers.
  - Think the podcast series has no effect on their relationships with peers.
5. Which of the following statements best describes the ideal length of podcasts?
  - Podcasts around 30 min in length are preferred by most students.
  - Podcasts longer than 45 min are preferred for in‐depth discussions.
  - Episodes shorter than 15 min are considered too brief for meaningful content.
  - Students have varying preferences regarding the length of podcast episodes.

### Recommended further reading

1. Girard E, Punch A, Jimenez Y. Framework for a radiography student podcast. J Med Radiat Sci 2024. 10.1002/jmrs.759.PMC1117702938282522
2. Karing C. The efficacy of online mindfulness‐based interventions in a university student sample: Videoconference‐ or podcast‐delivered intervention. Appl Psychol Health Well Being 2023;15:740–756. 10.1111/aphw.12408.36214182
3. Thomas H, Koch GGV. Clinical preparedness programme as perceived by first‐year diagnostic radiography students in South Africa. J Med Radiat Sci 2024;71:63–71. 10.1002/jmrs.740.PMC1092094737942815

## Answers

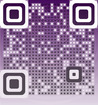

Scan this QR code to find the answers.

